# Distilling Big Data: Refining Quality Information in the Era of Yottabytes

**DOI:** 10.1155/2015/453597

**Published:** 2015-10-01

**Authors:** Sivaraman Ramachandramurthy, Srinivasan Subramaniam, Chandrasekeran Ramasamy

**Affiliations:** ^1^Anna University, Regional Office, Madurai 625007, India; ^2^Department of Computer Science and Engineering, Anna University, Regional Office, Madurai 625007, India; ^3^Department of Computer Science and Engineering, Annamalai University, Chidambaram 608002, India

## Abstract

Big Data is the buzzword of the modern century. With the invasion of pervasive computing, we live in a data centric environment, where we always leave a track of data related to our day to day activities. Be it a visit to a shopping mall or hospital or surfing Internet, we create voluminous data related to credit card transactions, user details, location information, and so on. These trails of data simply define an individual and form the backbone for user-profiling. With the mobile phones and their easy access to online social networks on the go, sensor data such as geo-taggings and events and sentiments around them contribute to the already overwhelming data containers. With reductions in the cost of storage and computational devices and with increasing proliferation of Cloud, we never felt any constraints in storing or processing such data. Eventually we end up having several exabytes of data and analysing them for their usefulness has introduced new frontiers of research. Effective distillation of these data is the need of the hour to improve the veracity of the Big Data. This research targets the utilization of the Fuzzy Bayesian process model to improve the quality of information in Big Data.

## 1. Introduction

Big Data systems are the eventual outcomes of today's data centric world. In this 21st century, we cannot imagine passing a single day without the touch of Internet. In fact the World Wide Web, online social networks, and extensive computerization of our everyday activities have put forth new phenomena in managing the data thrown out by these processes. By virtue of its multidimensional nature, comprising volume, velocity, variety, variability, and veracity (5Vs), Big Data poses serious research issues and its complexity is always in the increasing trend. The beauty of Big Data is the fact that it incorporates all types of data, not just pictures, texts, sounds, emails, and so forth, but also the inputs and outputs from sensors of any kind. Also these data can be either unstructured type arising out of social media transactions or even multistructural type which is the outcome of interactions between people and machines. The architecture of Big Data is different from the data warehousing solutions, as this architecture should cater to the 5Vs and should produce meaningful results [1].

According to a report by Forbes, the seed for the term Big Data was first sowed on the Communications of the ACM by Steve Bryson in the year 1999. This paper has highlighted the fact that advances in powerful computing threw out huge volumes of data and understanding from these data is a significant endeavour. Since then, the growth of Big Data occurred in a rapid manner with the increase in computing power and reduction in the cost of storage devices. Earlier, data capacity in the order of gigabytes (GB) looked big, but now terabytes (TBs) have become so common such that even desktop computers sport hard disk drives of 2TB. According to a recent study [2], the digital universe is doubling in its size every two years and the amount of data we generate and copy annually will become 44 trillion gigabytes or 44 zettabytes by 2020. More than 2 billion people and several million enterprises are doing their works online, thereby generating mass volumes of data. A single celeb-selfie tweet by Ellen DeGeneres who hosted the Oscars of 2014 was viewed 26 million times across the world within a short span of 12 hours, which shows the power of a single tweet to generate enormous data. In 2013, only 22% of the entire information processed in the digital universe was found to be useful if it has been properly tagged and a meagre 5% contained quality data. By 2020, the useful percentage of data may grow up to 35%, due to the contribution of data from embedded devices.

Parallel developments in the arena of Internet of Things (IoT) will accelerate the growth of Big Data with the sensors in mobile phones and other handheld devices pumping their own contributions [3]. New data sources leading to novel methodologies for data distilling and applying the outcomes to business processes so as to reap profits have become the cliché of successful businesses across the world. In short, smart work has outpaced the concept of hard work and proper data distillation techniques are needed to extract quality valuable data from the data dumps.

The people of today's Internet age have become more and more conscious about the markets. It has become increasingly tough to sell products to them and conventional marketing methodologies have become obsolete. In the financial sector, decision making has been revolutionized. Predictive analysis has entered into new dimensions about the functioning of stock markets and can provide a detailed insight about various investment portfolios. On the healthcare front, Big Data can help you arrive at tailor made solutions based on your past medical history. The new path breaking strategies in human genome mapping with Big Data tools, wherein genes are being mapped to the diseases as per the earlier medical records, can help doctors pinpoint the actual medicine to be administered.

## 2. Social Sensing

Social sensing is the new technology of human centric sensing, wherein the sensors are attached to the humans in a wearable form or use the mobile devices like cellphones which carry the sensors like GPS, camera, and so forth. As these mobile devices are capable of automatically connecting to the Internet, the data sensed by them can be accessed in no time across the connected applications and devices. Numerous applications like Cabsense, Bikenet, and Cenwits use the social sensing for their operations. In the field of healthcare, this concept has created wonders in continuously monitoring patient's vital data, emergency response, prediction about diseases, lifestyle effect on health, and so forth. Social sensing can be either voluntary/intentional or involuntary/unintentional. The former involves the participation from the user to identify the symptoms and causes whereas the latter does not have any user interaction and can happen without the knowledge of the user. Examples of user assisted sensing include marking the routes with more traffic and spotting the litter. The unassisted social sensing utilizes the built-in GPS of the hand held smart devices to automatically geo-tag the user and can use accelerometer to detect motion, and so forth. Reporting of natural calamities and civil unrest, on Twitter, Facebook, and YouTube, provides a global forum for sharing data on real time basis. Several research attempts are being made to understand the functioning of these online social sensing applications in propagation of information, characteristics, and tipping points.

## 3. Literature Review

**Figure 1:**
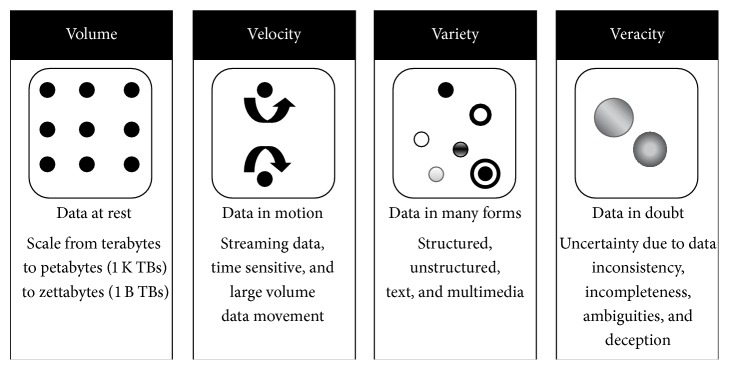
4Vs of Big Data.

With rapid decrease in the storage costs and dramatic increase in the computational capabilities, the size of the data is no longer a heinous factor and the quality of the data under study has become the primary concern. In one study by IBM, it was found that US $3.1 trillion was lost every day due to poor data quality and the survey has shown that 27% of the respondents were not sure about their own data. Since majority of the data come from anonymous unverified and untagged sources, the quality of the data needs to be flagged before it is used in any task [4]. Until recently, the Big Data has been characterized only by 3Vs, namely, volume, velocity, and variety, and the content looks more like a soup with the content providers scooping out and dumping them into information systems [5]. But with the introduction of data quality as a key factor, the fourth dimension of Big Data, namely, veracity, came into the picture which focusses on Information Quality (IQ) [6]. However big the quantity of data is, it will not be of any use if it does not have any meaningful extractable information and this research aims at providing a solution to the problem of data quality. Uncertainty in the data leads to unreliable information. This issue of uncertainty may be broadly classified into three heads, namely, process uncertainty, data uncertainty, and model uncertainty. The examples for process uncertainty are uncertain travel times in a GPS based tracking environment, yield of semiconductors, and so forth, and the data uncertainty involves inconsistency, ambiguity, incompleteness, conflicting data, and so forth. Model uncertainty comes into picture in the event of prediction and forecasting services where all models are approximations only. The data quality can generally be grouped under several categories like accuracy, believability, reputation, objectivity, factuality, consistency, freedom from bias, correctness, and unambiguousness. But with the introduction of uncertainty, all these factors have to be revisited to evolve a perfect working solution [7–11].  Figure 1 shows the 4Vs of Big Data as proposed by IBM [11].

Meeker and Hong [12] have discussed the opportunities and challenges in the Big Data related to the warranty and insurance claims in a typical industrial environment. With the technological advancements, leading to the implantation of sensors within the products, we can easily measure various parameters like use rate, system load, and environmental factors from these embedded sensors. The concepts represented in this research refer to the Internet of Things and the data are referred to as the SOE (System Operation/Environmental) Data and they can be well used to analyse the reliability issues. Locomotive engines, aircraft engines, power distribution transformers, CT scan devices, wind turbines, and solar energy power invertors are some examples where such sensor data are available to predict the reliability of the system.

Uddin et al. [13] extensively analysed the social sensing and have developed algorithms for improving the source selection in social networks like Twitter, Flicker, and so forth. These social networks provide unprecedented opportunities to develop sensor applications where humans either act as sensors or play as sensor operators by posting the observations or measurements in an online medium. As the observations posted by one user are available to other users, any wrong or faulty information by one user may pass on to others since there is no mechanism to verify its correctness. Baseless rumours that will mislead the facts may also propagate in these online networks thereby creating bias. Uddin et al. have found that social sensing brings in new type of inaccuracy called unknown dependence between the sources which affects the creditability of data. They have adopted the concept of diversifying the sources to extract quality information. However, their technique used the follow relationships in the Twitter as a method of measurement and does not focus on the geographic locations and community they belong to in the process of assessment.

Maximum likelihood estimation (MLE) method [14] was adopted to find the accuracy of the participants and this technique utilizes the Cramer-Rao bound for estimation purposes. This method provides an insight into participants reliability without knowing their reliability a priori. Fisher information was initially computed by using the asymptotic normality of MLE. Though satisfactory results were obtained, a tighter Cramer-Rao bound can be derived to measure the truthfulness of the variable.

Aggarwal and Abdelzaher [15] discussed in detail the social sensing applications in the web like Google latitude, Citysense, Macrosense, Wikitude, Green GPS, and so forth. With the development of miniaturized sensor technology, sensors can even be embedded in the cloth fabric and the user can wear the attire without any obstruction or protrusion. 3G/4G mobile wireless networks have virtually bridged the bandwidth performance between wired and wireless networks. Online real time stream processing systems have also been developed to fetch and analyse the sensor data on the fly. But these real time systems bring in several issues like data compression, challenges in handling multiple data streams, data collection in mobile devices, and so forth. Also privacy and trust factors always collide with each other as one demands data fidelity and the other denies it. With the inroads created by Big Data, certain new dimension has also been put forth by Mishra and Sharma [16] by introducing the concept of value and an in-depth study has been made in the significant developments in the area of national development, industrial upgrades, interdisciplinary research, and better perception of the present scenarios of any industry. An interesting work carried out by Najafabadi et al. [17] has focussed on the importance of applying deep learning methods and evolving strategies to yield better results in the analysis of Big Data. They have introduced the concept of feeding a part of the available data corpus into deep learning algorithms and utilizing the remaining input data corpus for pattern recognition to extract data abstractions and representations. Thus the variety characteristic of Big Data, which focuses on the varied input data types for training the data representations and the target data source for generalizing the representations, becomes a problem of domain adaptation in deep learning. Their research has also explored the selection of criteria to provide useful semantic meaning.

The veracity of a Big Data environment depends on various factors, which include trustworthiness and expertise. These two factors are responsible for providing credible judgments about the quality of data. Objectivity, truthfulness, and credibility have been designated as the three main theoretical dimensions of veracity [18]. Various Big Data problems can be characterized by these dimensions based on quality.

## 4. System Design

One of the critical questions in the research of social sensing is about the reliability of the data collected by the humans. The process of contributing information is open and we never had any filtering mechanism to identify the reliability of the humans who act as sensors. Trust analysis of social sensing data is still an underdeveloped area that needs new techniques to improve reliability and enhance quality of information. This paper aims at creating a suitable framework to measure the reliability of data sensing sources and also to study the exactness of their responses. This approach is based on the Fuzzy Bayesian Interpretation model [19–23] where the data is processed on the server side using software only and no specialized hardware is required. Though this paper has addressed the explosion of data and has covered in depth the size of zettabytes and yottabytes, this research has utilized the simulation of an information network comprising “*n*” number of sources and assertions. A network model based approach can be visualised for solving the reliability issues, with the nodes representing the unreliable information and the links representing the abstract relations. Let there be sources *S*_1_, *S*_2_, *S*_3_,…, *S*_*s*_, who collectively affirm information *C*_1_, *C*_2_, *C*_3_,…, *C*_*c*_. If these sources and assertions are made to represent the nodes, then the links between the corresponding source nodes and the assertion nodes are represented by *C*_*ij*_. Then the creditability of the sources is given by Cred (*S*_*i*_) and that of the assertion given by Cred (*C*_*j*_).

## 5. Methodology

### 5.1. Bayes Interpretation

We propose to use the Bayesian interpretation to rank the various sources providing information based on the creditability values. The statement of Bayes' theorem is

(1) $$\begin{array}{c} p\left(h\;|\;e\right)\propto p\left(e\;|\;h\right)p\left(h\right), \end{array}$$

where *p*(*h*∣*e*) and *p*(*e*∣*h*) are conditional probabilities. For example, if *p*(*e*∣*h*) is the probability of *e* based on the condition of *h*, according to Bayes rule, whenever an evidence of *e* is received, *p*(*h*) has to be updated by *p*(*h*∣*e*). We have to multiply the prior probability *p*(*h*) by the prior likelihood *p*(*e*∣*h*) followed by the normalization such that all probabilities sum to 1. The resulting posterior probability *p*(*h*∣*e*) is the revised probability assignment for *h*. Therefore, the new probability of *h* is proportional to its original probability, multiplied by the likelihood of evidence *e* given *h* [24].

**Figure 2:**
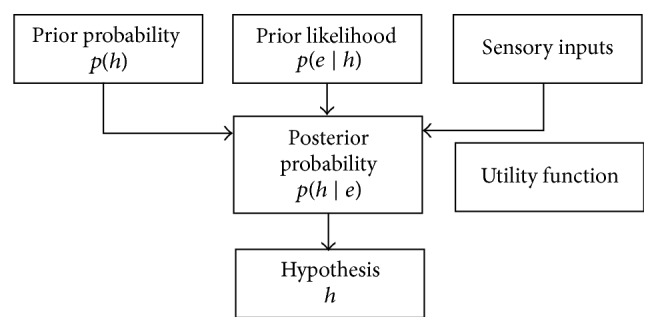
Typical Bayesian model.

A Bayesian model can well be explained by using the following template and it forecasts an outcome through four factors, namely, prior probabilities, prior likelihoods, sensory input, and the utility function. This shows that the proposed model is deterministic and it has been depicted in Figure 2. However certain sensory malfunctions, internal noise leading to corruption can be the basis for exceptions. Certain models also replace the expected utility maximisation with probability matching leading to a nondeterministic process.

A Bayesian interpretation proposed by Dong Wang et al. [25] involves the evaluation using multiple sources *S*_1_, *S*_2_,…, *S*_*j*_ with assertions *C*_*j*_. Then according to the Bayes theorem,

(2) $$\begin{array}{c} P\left(C_{j}^{t}\;|\;S_{i1}C_{j},S_{i2}C_{j},\ldots ,S_{ik}C_{j}\right)=\frac{P\left(S_{i1}C_{j},S_{i2}C_{j},\ldots ,S_{ik}C_{j}\;|\;C_{j}^{t}\right)}{P\left(S_{i1}C_{j},S_{i2}C_{j},\ldots ,S_{ik}C_{j}\right)}P\left(C_{j}^{t}\right). \end{array}$$

Here an implicit assumption is made that the probability of a source making any assertion in low and no change in posterior probability can be made from the nonavailability of claim. This shows that only existing claims are considered. Assuming, for any given assertion, the probability that any two sources claiming the same status are independent, then the equation can be rewritten as follows:

(3) $$\begin{array}{c} P\left(C_{j}^{t}\;|\;S_{i1}C_{j},S_{i2}C_{j},\ldots ,S_{ik}C_{j}\right)=\frac{P\left(S_{i1}C_{j}{\;|\;C}_{j}^{t}\right),\ldots ,P\left(S_{ik}C_{j}\;|\;C_{j}^{t}\right)}{P\left(S_{i1}C_{j},S_{i2}C_{j},\ldots ,S_{ik}C_{j}\right)}P\left(C_{j}^{t}\right). \end{array}$$

If the change in posterior probability is found to be small and this case arises when evidence is collected from several unreliable sources,

(4) $$\begin{array}{c} \frac{P\left(S_{ik}C_{j}\;|\;C_{j}^{t}\right)}{P\left(S_{ik}C_{j}\right)}=1+\delta_{ikj}^{t}. \end{array}$$

Similarly,

(5) $$\begin{array}{c} \frac{P\left(S_{ik}C_{j}\;|\;C_{j}^{f}\right)}{P\left(S_{ik}C_{j}\right)}=1+\delta_{ikj}^{f}, \end{array}$$

where

(6) $$\begin{array}{c} \left|1+\delta_{ikj}^{t}\right|\ll 1,\\ \left|1+\delta_{ikj}^{f}\right|\ll 1. \end{array}$$

Hence the equation can be rewritten as

(7) $$\begin{array}{c} P\left(S_{i1}C_{j},S_{i2}C_{j},\ldots ,S_{ik}C_{j}\right)\approx \overset{Kj}{\underset{k=1}{\prod }}P\left(S_{ik}C_{j}\right). \end{array}$$

Substituting the same in (2) we get

(8) $$\begin{array}{c} P\left(C_{j}^{t}\;|\;S_{i1}C_{j},S_{i2}C_{j},\ldots ,S_{ik}C_{j}\right)=\frac{P\left(S_{i1}C_{j}\;|\;C_{j}^{t}\right),\ldots ,P\left(S_{ik}C_{j}\;|\;C_{j}^{t}\right)}{P\left(S_{i1}C_{j}\right),\ldots ,P\left(S_{ik}C_{j}\right)}P\left(C_{j}^{t}\right), \end{array}$$

and rewriting it gives the following:

(9) $$\begin{array}{c} P\left(C_{j}^{t}\;|\;S_{i1}C_{j},S_{i2}C_{j},\ldots ,S_{ik}C_{j}\right)=\frac{P\left(S_{i1}C_{j}\;|\;C_{j}^{t}\right)}{P\left(S_{i1}C_{j}\right)}\times \frac{P\left(S_{ik}C_{j}\;|\;C_{j}^{t}\right)}{P\left(S_{ik}C_{j}\right)}\times P\left(C_{j}^{t}\right). \end{array}$$

Substituting from (7),

(10) $$\begin{array}{c} P\left(C_{j}^{t}\;|\;S_{i1}C_{j},S_{i2}C_{j},\ldots ,S_{ik}C_{j}\right)=P\left(C_{j}^{t}\right)\overset{Kj}{\underset{k=1}{\prod }}\left(1+\delta_{ikj}^{t}\right)=P\left(C_{j}^{t}\right)\left(1+\overset{Kj}{\underset{k=1}{\sum }}\delta_{ikj}^{t}\right). \end{array}$$

As higher orders of *δ*_*ikj*_^*t*^ can be neglected,

(11) $$\begin{array}{c} \frac{P\left(C_{j}^{t}\;|\;S_{i1}C_{j},S_{i2}C_{j},\ldots ,S_{ik}C_{j}\right)-P\left(C_{j}^{t}\right)}{P\left(C_{j}^{t}\right)}=\overset{Kj}{\underset{k=1}{\sum }}\delta_{ikj}^{t},\\ \delta_{ikj}^{t}=\frac{P\left(S_{ik}C_{j}\;|\;C_{j}^{t}\right)-P\left(S_{ik}C_{j}\right)}{P\left(S_{ik}C_{j}\right)}. \end{array}$$

### 5.2. Creditability of Sources

Let *S*_*i*_ be the source of information making claims Claims_*i*_ and *j*_*k*_ denotes the *k*th claim of Claims_*i*_ and |Claims_*i*_| = *L*_*i*_. By the Bayes theorem,

(12) $$\begin{array}{c} P\left(S_{i}^{t}\;|\;S_{i}C_{j1},S_{i}C_{j2},\ldots ,S_{i}C_{jL}\right)=\frac{P\left(S_{i}C_{j1},S_{i}C_{j2},\ldots ,S_{i}C_{jL}\;|\;S_{i}^{t}\right)}{P\left(S_{i}C_{j1},S_{i}C_{j2},\ldots ,S_{i}C_{jL}\right)}P\left(S_{i}^{t}\right). \end{array}$$

As earlier assuming the conditional independence,

(13) $$\begin{array}{c} P\left(S_{i}^{t}\;|\;S_{i}C_{j1},S_{i}C_{j2},\ldots ,S_{i}C_{jL}\right)=\frac{P\left(S_{i}C_{j1}\;|\;S_{i}^{t}\right),\ldots ,P\left(S_{i}C_{jL}\;|\;S_{i}^{t}\right)}{P\left(S_{i}C_{j1},S_{i}C_{j2},\ldots ,S_{i}C_{jL}\right)}P\left(S_{i}^{t}\right). \end{array}$$

Since the change in posterior probability arising out of any single claim is negligible,

(14) $$\begin{array}{c} \frac{P\left(S_{i}C_{jk}\;|\;S_{i}^{t}\right)}{P\left(S_{i}C_{jk}\right)}=1+\eta_{ijk}^{t}, \end{array}$$

where |*η*_*ijk*_^*t*^ | ≪1.

Hence,

(15) $$\begin{array}{c} \frac{P\left(S_{i}^{t}\;|\;S_{i}C_{j1},S_{i}C_{j2},\ldots ,S_{i}C_{jL}\right)-P\left(S_{i}^{t}\right)}{P\left(S_{i}^{t}\right)}=\overset{Li}{\underset{k=1}{\sum }}\eta_{ijk}^{t},\\ \eta_{ijk}^{t}=\frac{P\left(S_{i}C_{jk}\;|\;S_{i}^{t}\right)-P\left(S_{i}C_{jk}\right)}{P\left(S_{i}C_{jk}\right)}. \end{array}$$

### 5.3. Iterative Algorithm

In the preceding sections, posterior probability was derived based on the assumption that either an assertion is true or the source is truthful and they were derived from *δ*_*ikj*_^*t*^ and *η*_*ijk*_^*t*^:

(16) $$\begin{array}{c} P\left(S_{i}C_{j}\;|\;C_{j}^{t}\right)=\frac{P\left(S_{i}C_{j},C_{j}^{t}\right)}{P\left(C_{j}^{t}\right)}, \end{array}$$

where

(17) $$\begin{array}{c} P\left(S_{i}C_{j}\;|\;C_{j}^{t}\right)=P\left(S_{i}\;speaks\right)P\left(S_{i}\;Claims\;C_{j}\;|\;S_{i}\;speaks\right)P\left(C_{j}^{t}S_{i}\;speaks,S_{i}\;Claims\;C_{j}\right). \end{array}$$

Since the ground truth is unknown, the probability is estimated by considering the best information we have which is *P*(*S*_*i*_^*t*^∣*S*_*i*_ *C*_*j*1_, *S*_*i*_ *C*_*j*2_,…, *S*_*i*_ *C*_*jL*_). Thus,

(18) $$\begin{array}{c} P\left(S_{i}C_{j},C_{j}^{t}\right)=\frac{P\left(S_{i}\;speaks\right)P\left(S_{i}^{t}\;|\;S_{i}C_{j1},S_{i}C_{j2},\ldots ,S_{i}C_{jL}\right)}{C}. \end{array}$$

Substituting this into the above equations we get

(19) $$\begin{array}{c} P\left(S_{i}C_{j},C_{j}^{t}\right)=\frac{P\left(S_{i}\;speaks\right)P\left(S_{i}^{t}\;|\;S_{i}C_{j1},S_{i}C_{j2},\ldots ,S_{i}C_{jL}\right)}{Ctrue}. \end{array}$$

Similarly,

(20) $$\begin{array}{c} P\left(S_{i}C_{j}\right)=\frac{P\left(S_{i}\;speaks\right)}{C}. \end{array}$$

Therefore by rearranging we get

(21) $$\begin{array}{c} \delta_{ikj}^{t}=\frac{P\left(S_{ik}C_{j}\;|\;C_{j}^{t}\right)-P\left(S_{ik}C_{j}\right)}{P\left(S_{ik}C_{j}\right)},\\ \delta_{ikj}^{t}=\frac{P\left(S_{i}^{t}\;|\;S_{i}C_{j1},S_{i}C_{j2},\ldots ,S_{i}C_{jL}\right)}{Ctrue/c}-1. \end{array}$$

If we consider the fractions of all true assertions to the true assertions total as prior probability, for a source to be truthful, *P*(*S*_*i*_^*t*^), then the above equation can be rewritten as

(22) $$\begin{array}{c} \delta_{ikj}^{t}=\frac{P\left(S_{i}^{t}\;|\;S_{i}C_{j1},S_{i}C_{j2},\ldots ,S_{i}C_{jL}\right)}{P\left(S_{i}^{t}\right)}-1. \end{array}$$

Substituting for *δ*_*ikj*_^*t*^ we get

(23) $$\begin{array}{c} \frac{P\left(C_{j}^{t}\;|\;S_{i1}C_{j},S_{i2}C_{j},\ldots ,S_{ik}C_{j}\right)-P\left(C_{j}^{t}\right)}{P\left(C_{j}^{t}\right)}=\overset{Kj}{\underset{i=1}{\sum }}\frac{P\left(S_{i}^{t}\;|\;S_{i}C_{j1},S_{i}C_{j2},\ldots ,S_{i}C_{jL}\right)-P\left(S_{i}^{t}\right)}{P\left(S_{i}^{t}\right)}. \end{array}$$

Similarly it can be proved that

(24) $$\begin{array}{c} \eta_{ijk}^{t}=\frac{P\left(S_{i1}C_{j},S_{i2}C_{j},\ldots ,S_{ik}C_{j}\right)}{P\left(C_{j}^{t}\right)}-1,\\ \frac{P\left(S_{i}^{t}\;|\;S_{i}C_{j1},S_{i}C_{j2},\ldots ,S_{i}C_{jL}\right)-P\left(S_{i}^{t}\right)}{P\left(S_{i}^{t}\right)}=\overset{Li}{\underset{j=1}{\sum }}\frac{P\left(C_{j}^{t}\;|\;S_{i1}C_{j},S_{i2}C_{j},\ldots ,S_{ik}C_{j}\right)-P\left(C_{j}^{t}\right)}{P\left(C_{j}^{t}\right)}. \end{array}$$

Comparing the above equations we derive the creditability rank scores Rank (*S*_*i*_) and creditability rank assertions Rank (*C*_*j*_) using the iterative fact finding method:

(25) $$\begin{array}{c} Rank\;C_{j}=\frac{P\left(C_{j}^{t}\;|\;S_{i1}C_{j},S_{i2}C_{j},\ldots ,S_{ik}C_{j}\right)-P\left(C_{j}^{t}\right)}{P\left(C_{j}^{t}\right)}=\underset{k\in {Sources}_{j}}{\sum }Rank\;S_{k},\\ Rank\;S_{i}=\frac{P\left(S_{i}^{t}\;|\;S_{i}C_{j1},S_{i}C_{j2},\ldots ,S_{i}C_{jL}\right)-P\left(S_{i}^{t}\right)}{P\left(S_{i}^{t}\right)}=\underset{k\in {Claims}_{i}}{\sum }Rank\;C_{k}. \end{array}$$

When the creditability ranks are found such that they satisfy these equations, along with the assumption that with the prior probability assertion is true being initialized to *p*(*t*/*a*) = *C*true/*c*, it gives us the

(26) $$\begin{array}{c} P\left(C_{j}^{t}\;|\;network\right)=p_{a}^{t}\left(Rank\left(C_{j}\right)+1\right). \end{array}$$

Similarly if *p*_*s*_^*t*^ is the prior probability of a chosen source telling truth then

(27) $$\begin{array}{c} P\left(S_{i}^{t}\;|\;network\right)=p_{s}^{t}\left(Rank\left(S_{i}\right)+1\right). \end{array}$$

Hence the abovementioned Bayesian analysis provides the platform for finding the probability for each individual source *S*_*i*_ and assertions *C*_*j*_ to be true.

### 5.4. Fuzzy Bayesian Inference

A fuzzy posterior approximator has been applied to yield

(28) $$\begin{array}{c} F\left(\theta \;|\;x\right)=\overset{m}{\underset{j=1}{\sum }}p_{j}\left(\theta \right)C_{j}^{i}\left(x\;|\;\theta \right), \end{array}$$

where

(29) $$\begin{array}{c} C_{j}^{i}\left(x\;|\;\theta \right)=\frac{C_{h}G\left(x\;|\;\theta \right)}{\sum_{i=1}^{m}\int_{d}^{}G\left(x\;|\;u\right)p_{i}\left(u\right)C_{h}i\;du}. \end{array}$$

Adding fuzzy rules and weights we get

(30) $$\begin{array}{c} F\left(\theta \;|\;x\right)=\frac{\sum_{i=1}^{m}w_{F}a_{F}\left(\theta \right)V_{F},iC_{F},i}{\sum_{i=1}^{m}w_{F}a_{F}\left(\theta \right)V_{F},i}, \end{array}$$

where the weights *w*_*F*_ and then part set volumes *V*_*F*_ are given by

(31) $$\begin{array}{c} w_{F}=w_{g},w_{h},k,\\ V_{F}=V_{g},V_{h},k. \end{array}$$

## 6. Results and Discussions

**Figure 3:**
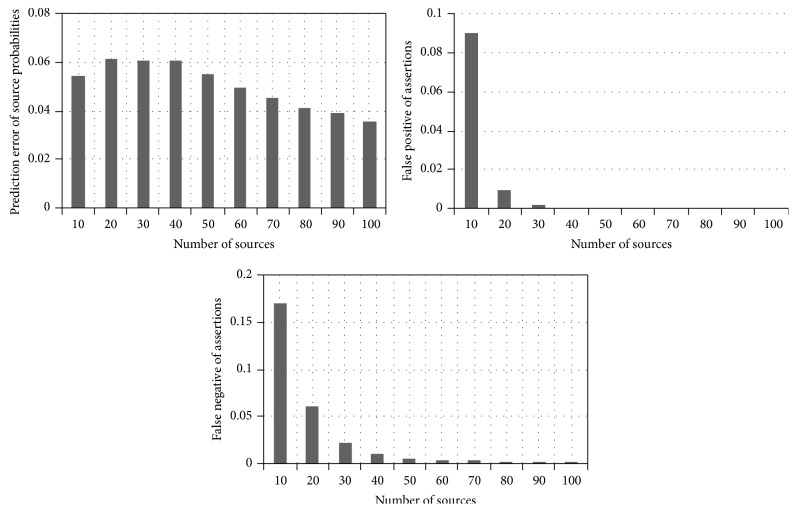
Prediction accuracy versus varying number of sources.

**Figure 4:**
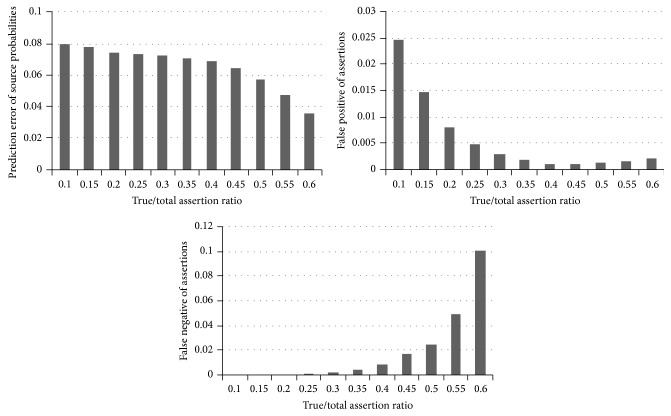
Prediction accuracy versus varying number of assertions.

**Figure 5:**
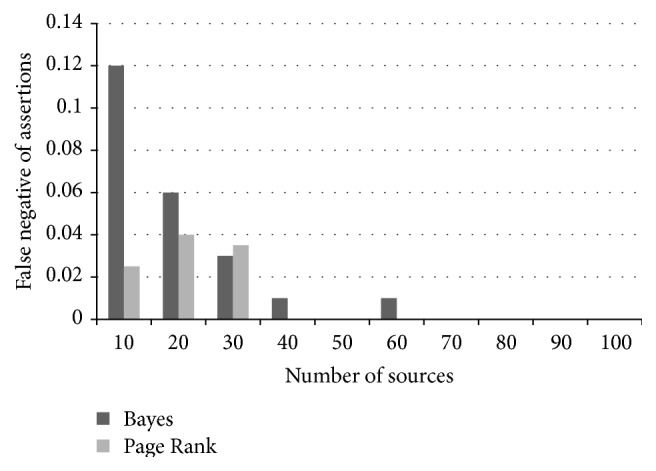
Prediction comparison.

We carried out the experiments using MATLAB 2013, by simulating the source and assertion information. We evaluated the results by using extensive simulations by generating up to 100 sources and 1000 assertions. Experiments were carried out to verify the accuracy and correctness of the probability that a given source is truthful or the given assertion is found truthful by the proposed Fuzzy Bayesian Scheme of interpretations. This technique is then compared with the existing methods like Page Rank and the results are tabulated. To test the results we randomly generated the source and assertions and partitioned these assertions into true and false. *P*_*i*_ is the random probability assigned to each source *S*_*i*_ generating *L*_*i*_ claims. Every claim has *P*_*i*_ probability of being true and 1 − *P*_*i*_ probability of being false. We compared the proposed Fuzzy Bayesian Interpretation scheme with that of the Page Rank algorithm. For every source correctness probability distribution, the results were averaged over 100 datasets in a time series. The number of true and false assertions was fixed as 1000 and the claims per source are set as 100. The total number of sources is initially set as 10 and is gradually moved towards 100. The prediction accuracy is shown in Figure 3. It was observed that the false positives and false negatives decreased with the increase in the number of sources. Relatively small error percentage of 5% to 8% was encountered by the source correctness probability. This probability is very accurate in the order of 0.1, when the large number of sources is considered. Figure 3 shows the graph between the prediction accuracy and the varying number of sources for prediction error of source probabilities, false positive of assertions, and false negative of assertions. We varied the count of true assertions to the overall total of assertions in the network and fixed the total number of assertions as 3000 and the number of sources as 30. The ratio of true to total assertions was changed from 0.2 to 0.8. It has been observed that the error of source correctness probability prediction decreases with the increase in the ratio of true assertions. Alternatively, the numbers of false negatives increase, as more and more true assertions are misclassified as false.  Figure 4 shows the graph between the prediction accuracy and the varying number of assertions for prediction error of source probabilities, false positive of assertions, and false negative of assertions. At last, these results are compared with the Page Rank algorithm and the results are displayed as graph. The results show that Fuzzy Bayesian Interpretation outperforms the Page Rank algorithm for the given number of sources and the same has been shown in Figure 5. The observed results have confirmed the advantages of Fuzzy Bayesian approach over the conventional Bayesian analysis [24] in comparison with Page Rank.

## 7. Conclusion

In this research, an analysis of information networks was presented using the Fuzzy Bayesian approach and it has been successfully verified to assess the creditability of sources and assertions. Though several rank based algorithms are present, this paper converted the rank to a fuzzy probability method to find the truthfulness of a source and its assertion. With the ever ending increase in the smart devices under the category “Internet of Things,” today's businesses have understood the importance of Big Data as they have opened up new frontiers in the field of business intelligence. Traditionally business intelligence adopted a revolutionary approach by bringing in the Big Data in Hadoop based environments and deriving the reporting, modelling, and prediction systems. Now, the businesses are moving towards evolutionary approaches or even hybrid approaches that use the combination of techniques of Big Data and business intelligence. With the Big Data becoming the primary input for business intelligence tools, social sentiment analysis and usage patterns form the backbone to market any product across the world leading to the concept of social sensing. This is a rapidly developing technology and, with the ever increasing mobile devices proliferation, this technique gains momentum. With the miniaturization of the sensors and the ease with which they are embedded into mobile devices, social sensing will definitely have a widespread acceptance and usage. Though the mobile device and the associated sensors possess high degree of reliability, when humans act as the operators of these sensors, the reliability becomes a question mark. Hence several researches similar to the one presented in this paper are being conducted across the world to improve the veracity of the Big Data. With the explosion of Cloud Computing, the concept of Cloud supported analytics has already evolved and is becoming multidimensional [26]. This new paradigm enables the infrastructure to scale up or down dynamically, adapting the system to the actual demand. Analytics as a Service (AaaS) or Big Data as a Service (BDaaS) is another budding area which is quite interesting and can take us towards ultimatum. Though the present study has incorporated only the Fuzzy Bayesian and Page Rank algorithms, future studies can be done using several advanced techniques like Maximum Likelihood Estimation and Expectation Maximisation to bring in more reliability in an undependable system.
